# *Dirofilaria immitis* prevention messaging: Knowledge and attitudes of students from North America attending the Ross University School of Veterinary Medicine, St Kitts

**DOI:** 10.1002/vro2.32

**Published:** 2022-04-09

**Authors:** Kaitlyn Jonk, Mary Mauldin Pereira, Jennifer Ketzis, Anne Conan

**Affiliations:** ^1^ Department of Biomedical Sciences Ross University School of Veterinary Medicine Basseterre, St. Kitts and Nevis West Indies; ^2^ Department of Clinical Sciences Ross University School of Veterinary Medicine Basseterre, St. Kitts and Nevis West Indies; ^3^ Center for Applied One Health Research and Policy Advice, Kowloon City University of Hong Kong Hong Kong SAR China

## Abstract

**Background:**

The expansion of vector‐borne diseases is considered to be a threat to pet health. Some diseases such as heartworm disease have effective individual prevention methods; however, population‐level disease control is limited by the lack of treatment compliance by pet owners. Veterinarians have a primary role in increasing compliance by educating owners on the appropriate prevention measures. Veterinary educational approaches targeting prevention strategies could strengthen prevention messaging at a clinic level.

**Methods:**

A knowledge and attitude study was conducted with incoming Ross University School of Veterinary Medicine students as a preliminary assessment of this hypothesis.

**Results:**

Seventy‐three students were interviewed using a tested and standardised questionnaire during their first weeks and 38 answered the same questionnaire one year later. All of the participants had previous experience in a veterinary clinical setting. Knowledge about the disease was variable, usually higher in former veterinary technicians. Unfortunately, knowledge of heartworm prevention was low. In addition, willingness to share knowledge on disease prevention did not change even after one year in veterinary school.

**Discussion:**

These results suggest that additions within the veterinary and veterinary technician school curriculum may be required to improve knowledge about disease prevention and ultimately promote better communication with pet owners and veterinary clinical teams.

## INTRODUCTION

The geographical distribution of the parasite *Dirofilaria immitis* has dramatically expanded in the last few decades.^1^ While its endemic range was relatively restricted in the past, the local transmission of this parasite now occurs within all of the USA and is observed in southern Canada. This expansion makes heartworm infection a potentially significant health consideration for dog populations in North America. Two independent studies conducted by the American Heartworm Society (AHS) and the Companion Animal Parasite Council (CAPC) reported an increase of incidence by 15.3%–21.7% from 2013 to 2016 in the USA. These studies also reported an increase in the number of dogs being tested, while the proportion of animals receiving preventative care remained constant.^1^, ^2^ To date, the best measure to mitigate the spread of *D. immitis* is the regular use of anthelmintics, specifically macrocyclic lactones (MLs), targeting third and early fourth‐stage larvae. Increasing the number of dogs receiving MLs not only protects those dogs from heartworm infection but also decreases heartworm incidence in dogs not receiving regular administration of MLs.^3^ Clear and consistent health education messaging from veterinarians and the veterinary profession is required to improve owner compliance in the use of MLs.^4^ Enhancing client communication within the veterinary profession could be one solution to raise the understanding and compliance of pet owners.

On St Kitts, a Caribbean island and home to Ross University School of Veterinary Medicine (RUSVM), the highest prevalence of *D. immitis* occurs in a higher socio‐economic resident area that houses a large population of RUSVM students. Many of these students own both dogs and cats which have come from *D. immitis* endemic regions in North America and have worked in veterinary clinics, many of whom as veterinary technicians. A knowledge and attitude study was conducted to assess if students could be used to reinforce messaging from veterinary clinics with the goal of improving *D. immitis* prevention compliance.

## MATERIAL AND METHODS

### Study design

In September 2017, students newly enrolled in RUSVM veterinary and veterinary preparatory programmes were asked to participate in a study presented as focusing on an infectious agent in dogs. Students were interviewed during their first weeks on St Kitts. The name of the pathogen was not mentioned to prevent bias in the knowledge assessment. After signing the consent form, participants completed a standardised questionnaire. One year later (September 2018), the same questionnaire was asked to the same participants. This second survey occurred after they had completed a course in parasitology and before completing courses in pharmacology and small animal medicine. The participants' overall Grade Point Average (GPA) over the three semesters (Fall 2017, Spring 2018, Summer 2018) was retrieved from the registrar's office. The study was approved by the RUSVM Institutional Review Board (Protocol #17‐10‐XP).

### Questionnaire

The questionnaire included four parts. Part 1 gathered background information (seven questions) and collected data encompassing participant demographics, geographical origin, current home location, animal ownership and previous experience in veterinary settings. Part 2, knowledge (10 questions, most open ended), evaluated the participant knowledge on epidemiology, clinical presentation and prevention of heartworm. Part 3, about attitudes (17 questions), assessed the importance participants gave to *D. immitis* as a health issue for dogs and cats. Attitudes while living on St Kitts or living at home were asked separately. A five‐point Likert scale (from strongly disagree to strongly agree) was used for all questions except two. These two questions asked for recommendations on the prevention of the disease. The fourth part, practices (five questions), was provided only to dog owners. The questions focused on the use of preventative medicine, management of the dog and possible positive diagnosis of heartworm. The questionnaire was first tested with ten veterinary students further along the course to assure the questions’ understandability and consistency. The questionnaire is provided in Supporting Information 1.

### Data analyses

Data were collected using Epi Info™ software.^5^ Data analyses were performed using R software^6^ and consisted mainly of descriptive statistics.

Open‐ended questions with simple answers (e.g. ‘what is the scientific name of the agent’) were coded as a binary variable (1: correct, 0: not correct). Major spelling errors resulted in the answer being coded as incorrect. Three questions regarding knowledge of transmission, clinical signs and prevention of heartworm requested an essay‐type answer and were classified into four categories (0 = Absence of knowledge, 1 = Sparse knowledge, 2 = Basic knowledge, 3 = Above basic knowledge) (see Table 1). Knowledge and attitudes were described for each survey (2017 and 2018). The number of respondents to the section ‘Practice’ was low (*N* = 5 and 15 for the first and second surveys, respectively); therefore, these data are not presented in this paper.

**TABLE 1:** Coding of the open‐ended questions on knowledge of transmission, clinical signs and prevention of heartworm (*Dirofilaria immitis*) into ordinal variables (0–3)

	Transmission	Clinical signs	Prevention
None (0)	No answer given or the answer indicates a mode of transmission other than mosquito borne	Incorrect clinical sign(s) given and/or none of the clinical signs is in relation to heartworm	No answer given or the answer is not related to prevention of heartworm
Sparse knowledge (1)	Correct about mosquito vector but may also contain incorrect information regarding larval stages or transmission	At least one correct clinical sign given or at least two correct and one incorrect clinical signs given	Knowing a preventative of some form is given
Basic knowledge (2)	Correct about mosquito vector, disease transmission and larval involvement in cycle	At least two correct clinical signs given	Knowing a preventative is given either monthly or as a long‐term injection
More than basic knowledge (3)	Correct about mosquito vector, disease transmission and larval involvement in cycle with correct life stages (Microfilariae/L1 + L3) identified	More than two correct clinical signs given	Basic knowledge plus one or more of the following: naming two or more heartworm products or additional control methods or diagnostic methods prior to starting preventative

Knowledge and attitudes were compared between the first and second surveys using McNemar's test (for categorical variables) and paired Wilcoxon signed‐rank test (for ordinal variables). The answers to the knowledge and attitude sections were also compared between the different categories. For the first survey, variables of comparison encompassed: (i) the prior work position of the respondent while working in a veterinary clinic, (ii) the heartworm incidence status of the USA home state, the USA state of last year of residence and the veterinary clinic location state and (iii) the ownership of dogs outside of St Kitts. For the work position in the veterinary clinic, interns and shadows were classified as trainees and caretakers as veterinary assistants; if several functions were reported, the highest rank was kept (veterinary technician followed by veterinary assistant, office assistant and trainee).

The heartworm incidence status of a state was based on the heartworm incidence maps from the AHS (www.heartwormsociety.org/pet‐owner‐resources/incidence‐maps), which represent the number of cases reported by veterinary clinics to the AHS. A state that observed at least one area with a minimum of 26 cases by clinic was classified as a state with a high heartworm incidence. The rest of the states were considered as having a low incidence. The coding was performed using the 2007 AHS incidence map for hometown states and 2016 AHS incidence map for the last year of residence states and work states (Supporting Information 2). Locations including countries other than the USA as well as the USA territories (e.g. Puerto Ricco and the USA Virgin Islands) were excluded. Baseline data comparisons were performed using Fisher's exact test (categorical variables), Wilcoxon rank‐sum test (ordinal variables and GPA, two groups of comparison) and Kruskal–Wallis one‐way analysis of variance (ordinal variables, more than two groups of comparison). The threshold of significance was set with a *p*‐value of 0.05.

## RESULTS

In September 2017, 73 participants enrolled in the study. Thirty‐eight (52.1%) participated in the second survey in September 2018. The overall GPA of the second survey participants was not significantly different from the GPA of the non‐participants to the second survey (*p* = 0.2). Demographic data of the participants are described in Table 2. Students from the USA (*N* = 70, 95.9%) reported their hometown to be in one of 27 states or two different territories (Puerto Rico and the USA Virgin Islands). The highest frequency of home state was from the state of New York (*N* = 10). Out of the 73 respondents, 35.6% (*N* = 26) spent their last year in a state other than the state/country of the hometown. Almost all participants owned at least one animal back home (*N* = 68, 93.2%) comprising dogs (*N* = 59, 80.8%) and cats (*N* = 37, 50.7%). All students (*N* = 73) reported to have previously worked in a veterinary setting: 30 (41.1%) as a veterinary technician, 32 (43.8%) as a veterinary assistant, eight (15.1%) as an office assistant (administrative/receptionist/manager) and 25 (34.2%) as a trainee. Fifty‐nine (80.8%) participants worked more than 1 year in a veterinary clinic. Out of the 69 respondents who worked in the USA, 33 (47.8%) worked in a ‘high‐incidence state’ (AHS 2016 data). Knowledge and attitudes towards heartworm are summarised in Tables 3 and 4. Overall, knowledge improved over the year, but attitudes stayed the same. The participants tended to give advice to their relatives back home about the dog and/or the cat health.

**TABLE 2:** Description of demographic data of the knowledge, attitudes and practices survey respondents

		First survey	Second survey
Number of respondents		73	38
Gender^a^	Female	59 (81.9%)	30 (78.9%)
	Male	13 (18.1%)	8 (21.1%)
Age (in years)	Mean	23.3	23.9
	Interquartile range	22–24	23–24
	Range	21–33	22–28
Enrolled semester at Ross University School of Veterinary Medicine (parasitology course takes place during the second semester)	Vet prep	19 (26.0%)	–
	1	54 (74.0%)	0 (0%)
	2	–	2 (5.3%)
	3	–	11 (28.9%)
	4	–	25 (65.8%)

^a^ One participant did not answer the gender question during the first survey.

**TABLE 3:** Knowledge of heartworm (*Dirofilaria immitis*) among study participants and evolution of this knowledge between the first and second surveys

Question [correct answer]		Survey 1		Survey 2		*p*‐Value
Have you ever heard about heartworm disease before?		72 (98.6%)		38 (100%)		‐
How do you know about heartworm?						
	I have/had an animal who was diagnosed with heartworm	5 (6.8%)		2 (5.3%)		
	I know somebody whose animal was diagnosed with heartworm	18 (24.7%)		14 (36.8%)		
	I have/had an animal and the veterinarian educated me about heartworm	28 (38.4%)		26 (68.4%)		
	I worked in a veterinarian clinic	61 (83.4%)		31 (81.6%)		
	General (TV, …)	2 (2.7%)		1 (2.6%)		
Do you know the scientific name of the agent? (Yes)		9 (12.3%)		33 (86.8%)		<0.001^a^
	What is it? [*Dirofilaria immitis*]	6 (8.2%)		20 (52.6%)		<0.001^a^
Do you know which type of agent it is? (multiple choice) [Parasite]		60^b^ (83.3%)		38 (100%)		–
What is the main animal infected by this agent? [Dog/Canine]		65 (89.0%)		37 (97.4%)		
Does the agent infect other animals? (Yes)		53 (72.6%)		37 (97.4%)		–
	Cat listed (in main or second)	40 (54.8%)		38 (100%)		–
Can the agent infect humans?		14 (19.2%)		7^b^ (18.9%)		1 ^a^
How is the agent transmitted?						0.004^a^
	Direct contact	2 (1.4%)		0		
	Indirect contact (via environment)	11 (15.1%)		0		
	By an animal vector	49 (67.1%)		37 (97.4%)		
	Don't know or answered several categories	11 (15.1%)		1 (2.6%)		
Can you describe the transmission?						<0.001^c^
	0	31 (42.5%)		1 (2.6%)		
	1	33 (45.2%)		18 (47.4%)		
	2	9 (12.3%)		12 (31.6%)		
	3	0		7 (18.4%)		
Can you describe the clinical signs?						<0.001^c^
	0	31 (42.5%)		0		
	1	14 (19.2%)		7 (18.4%)		
	2	19 (26.0%)		17 (44.7%)		
	3	9 (12.3%)		14 (36.8%)		
Can you describe the method to prevent heartworm?						0.03^c^
	0	5 (6.8%)		2 (5.3%)		
	1	30 (41.1%)		7 (18.4%)		
	2	29 (39.7%)		25 (65.8%)		
	3	9 (12.3%)		4 (10.5%)		
How often should the prevention be given to *dogs*?						0.2^a^
	Daily	0		0		
	Weekly	0		0		
	Monthly	63 (87.5%)		37 (97.4%)		
	Every 2 months	0		0		
	Every 6 months	7 (9.6%)		1 (2.6%)		
	No opinion	2^b^ (2.7%)		0		
How often should the prevention be given to *cats*?						0.001^a^
	Daily	0		0		
	Weekly	0		0		
	Monthly	43 (58.9%)		33 (86.8%)		
	Every 2 months	2 (2.7%)		0		
	Every 6 months	0		1 (2.6%)		
	No opinion	28 (38.4%)		4 (10.5%)		

^a^ Comparison is performed by McNemar test.

^b^ One missing value.

^c^ Comparison is performed by paired Wilcoxon signed‐rank test.

**TABLE 4:** Attitudes towards heartworm (*Dirofilaria immitis*) among study participants and evolution of these attitudes between first and second surveys

	Survey 1		Survey 2		
	Median	IQR	Median	IQR	*p*‐Value
Heartworm is an important disease in *dogs* in USA/Canada^a^	5	5–5	5	5–5	0.2
Heartworm is an important disease in *cats* in USA/Canada^a^	4^b^	3–5	4	3–4	0.4
Heartworm is an important disease in *dogs* in St Kitts and Nevis^a^	5	4–5	5^b^	5–5	0.4
Heartworm is an important disease in *cats* in St Kitts and Nevis^a^	4	3–5	4^b^	3–5	0.1
Local dogs from St Kitts and Nevis are more resistant to heartworm infection compared to imported *dogs* ^a^	3	1–3	2	1–3	0.2
I advise my neighbour/my family/my friend, who have a *dog(s)* in USA/Canada to give it preventative against heartworm^c^	5	5–5	5	4.25–5	1
I give information on dog general health to my neighbour/my family/my friend, who have a *dog(s)* in USA/Canada^c^	4^b^	4–5	4	4–5	0.5
I advise my neighbour/my family/my friend who have a *dog(s)* in St Kitts to give it preventative against heartworm^c^	3^b^	3–5	5	2–5	0.2
I give information on dog general health to my neighbour/my family/my friend, who have a *dog(s)* in St Kitts^c^	3	2–4	3.5	2–5	0.2
I advise my neighbour/my family/my friend, who have a *cat(s)* in USA/Canada to give it preventative against heartworm^c^	3^b^	1–4	3^b^	2–5	0.01
I give information on cat general health to my neighbour/my family/my friend, who have a *cat(s)* in USA/Canada^c^	4	2–5	4^b^	3–5	0.7
I advise my neighbour/my family/my friend who have *a cat(s)* in St Kitts to give it preventative against heartworm^c^	3	1–3	3^b^	1–4	0.3
I give information on cat general health to my neighbour/my family/my friend, who have a *cat(s)* in St Kitts^c^	3	1–4	3	2–4	0.09
I have more worries about heartworm now that I am living in St Kitts^a^	3	2–4	3^d^	1–3	1

Abbreviation: IQR, interquartile range.

^a^ 1 = Strongly disagree, 2 = Moderately disagree, 3 = Neutral, 4 = Moderately agree, 5 = Strongly agree.

^b^ One missing value.

^c^ 1 = Never, 2 = Sometimes, 3 = Neutral, 4 = Often, 5 = Always.

^d^ Thirteen missing values.

During the first survey, more participants who lived the preceding year in a high‐incidence state (AHS 2016 data) reported having heard about heartworm because they knew somebody whose animal was diagnosed with heartworm (*N* = 13/34, 38.2%) compared to participants who lived in a low‐incidence state (*N* = 5/36, 13.9%; *p* Fisher's exact test = 0.03). Also, more veterinary technicians (*N* = 27/30, 90%) and veterinary assistants (*N* = 23/24, 95.8%) than trainees (*N* = 11/19, 57.9%) reported having heard about heartworm because they worked in a veterinary clinic (*p* Fisher's exact test = 0.003). Surprisingly, more participants working in low‐incidence states reported knowing the name of the agent (*N* = 8/35, 22.9%) compared to participants working in high‐incidence states (*N* = 1/33, 3.0%) (*p* Fisher's exact test = 0.003).

During the first survey, significant differences in knowledge and attitudes were mainly observed between the categories of former work positions in a veterinary setting. Veterinary technicians believed they knew the name of the agent (*N* = 7/29, 24.1%) more often than veterinary assistants (*N* = 2/24, 8.3%) and trainees (*N* = 0/19) (*p* Fisher's exact test = 0.03). They also more often knew that transmission was by a vector/intermediate host (*N* = 26/30, 86.7%) when compared to the 16 veterinary assistants (out of 24, 66.7%) and seven trainees (out of 19, 36.9%) (*p* Fisher's exact test = 0.002). Finally, estimated scores in transmission knowledge and clinical knowledge were different between veterinary technicians, veterinary assistants and trainees (Kruskal–Wallis one‐way analysis of variance = 0.005 and 0.02, respectively). Veterinary technicians obtained higher scores (median = 2 and 3, respectively) than trainees (median = 1 and 1) (Wilcoxon rank‐sum test = 0.01). However, the scores for prevention knowledge were not significantly different between groups (median of 2 for trainees, 2 for veterinary assistants and 3 for veterinary technicians; *p* Kruskal–Wallis one‐way analysis of variance = 0.4). All (*N* = 30/30, 100%) veterinary technicians knew the frequency of preventative administration versus 87.0% (*N* = 20/23) of veterinary assistants and 68.4% (*N* = 13/19) of trainees (*p* Fisher's exact test = 0.002). Interestingly, trainees reported more often than the other categories that they advised their relatives who have cats to give preventative against heartworm (median score: 4 vs. 3 for veterinary assistants and 1.5 for veterinary technicians; *p* Kruskal–Wallis one‐way analysis of variance = 0.002).

Other analyses indicated some logical outputs. Dog owners were more likely to report a safe way of prevention in dogs (a median 5 among 59 owners vs. a median 4.5 among 14 non‐owners, *p* Wilcoxon rank‐sum test = 0.01). Additionally, cat owners gave information on cat general health to their relatives who have a cat more often than non‐cat owners (median of 4 in 37 cat owners vs. 3 in 36 non‐cat owners).

## DISCUSSION

According to this study, incoming veterinary students have an intermediate level of knowledge of *D. immitis*. The participants knew already about the parasite or the disease but failed to give accurate details regarding the epidemiology, clinical presentation and prevention methods of the disease. These results were initially expected as the participants had just started their veterinary curriculum. However, demographic questions revealed that most of the participants were dog owners and came from areas in the USA where the parasite is endemic. Moreover, almost one‐quarter of the respondents knew a person owning a heartworm‐positive dog. One could expect that this background as dog owners and interest in veterinary medicine would give them some basic knowledge about the parasite and its prevention.

Somewhat worryingly, all of the participants reported having worked previously in veterinary settings. In these settings, the employees being the most in contact and communication with the pet owners are the veterinary assistants and the veterinary technicians.^7^ A previous study^8^ described these communication tasks as ‘worked the phones’, ‘worked intake’ (welcoming the owners and making the first care) and ‘worked discharge’ with this last task encompassing food, diet and prevention product advice. The discussion with owners during the animals’ discharge aims to advise about the health and welfare of the animal.^8^ Heartworm prevention messaging should be a component of this task. Unfortunately, our results show indirectly that the veterinary technicians and assistants do not have the appropriate knowledge to properly convey a prevention message.

Compliance with prevention protocols is low in pet owners. For example, one study in France indicated a low percentage of compliance in the deworming of cats (36%) and dogs (6%).^9^ Consistent and effective communication is shown to improve owner compliance and consequently pet health and welfare.^10^ Several methods are implemented in veterinary clinical settings to inform pet owners about the principal health issues (vaccination, antimicrobial resistance, parasites prevention). Unfortunately, to the best knowledge of the authors’, little published study has been conducted in evaluating the best methods to convey effective messages to pet owners, although a few studies have indicated the need to involve the owner in the decision‐making process by a two‐way communication and the need of an simple oral message.^11^, ^12^, ^13^ One study^14^ also highlighted the limited impact of posters. More research to understand the leverages of compliance in pet owners is needed. The role of the veterinary assistant and veterinary technician could, therefore, be crucial to improve pet owner knowledge and compliance by reinforcing a strong and correct health message.

In our study, the veterinary technicians were more knowledgeable about the transmission and clinical aspects of the disease than the other veterinary workers. However, they were not more knowledgeable in regards to medical prevention. In the USA, the veterinary technician degree is a 2‐year degree, with various theoretical and practical subjects, including infectious diseases (transmission and clinical signs).^8^ We would like to suggest that preventative measures and also therapeutic measures (to help with decision‐making and compliance) should be emphasised more within the curriculum to improve general knowledge on these topics.

The attitudes of the participants showed that they were generally keen to give advice. They usually considered heartworm as an important disease and moderately advised their relatives about dog and cat general health and preventatives against heartworm. We hypothesised that improving knowledge and communication skills during their first year of veterinary school would give them more confidence in advising their relatives. However, the attitudes of the participants did not change between the two surveys, even with most of the students attending a parasitology course (semester two) and a communication lab course (semester three: ‘Communication Lab—Veterinary consultation 1’). Three interpretations are possible for this absence of difference: overconfidence of the participant at the beginning of the study, a fear to convey an incorrect message or a lack of communication improvement. The lack of communication skills is known within the veterinary profession.^11^, ^15^, ^16^ Training communication skills in veterinary practices showed an improvement in the relations between veterinarians, clients and the decision‐making process.^16^ Communication has been integrated into veterinary curriculums; however, this topic remains a minor subject. Improving veterinary medical professionals’ ability to communicate should be a priority in veterinary and veterinary technician schools. This also could help with decreasing fear to convey an incorrect message. Further studies spanning a longer time frame and focusing on communication ability would be beneficial to improving communication education for future veterinarians.

The present study has some limitations. Firstly, the questionnaires were completed without interviewer input. Consequently, the interpretation of the open questions could be biased. When asking an open question, it is often recommended to ask the participant if they wish to add anything. The absence of recall from the interviewer left the interviewee to estimate the amount of information needed. Some of our participants may not have written all of the information/knowledge they had, thereby decreasing their knowledge scores. A bias of selection was also present. As the participation was open to all incoming students, those that decided to participate might have felt more confident answering a questionnaire regarding knowledge of an unknown infectious agent. This would explain why all the participants had a background in veterinary practice. Our population may therefore be biased towards veterinary technicians. Moreover, the enrolment was based only on a single incoming semester at RUSVM. The participants may not be representative of all RUSVM students and/or of all students enrolling in veterinary curriculums in the USA. The second survey presented a drop in participation and once again a selection bias: the second survey participants may have been performing better within the curriculum and hence were more willing to participate. This hypothesis is unlikely in consideration of the absence of difference in the GPA between the second survey participants and non‐participants. However, improvement in the knowledge between the first and second surveys should still be cautiously interpreted.

Following these unexpected results, we advise repeating the study within several veterinary schools in the USA to confirm our results. More research surrounding the impact of health messages in small animal veterinary settings should be conducted on heartworm and also other health issues.^14^ Adopting an approach combining classical prevention campaigns and innovative communication tools to inform the pet owner may improve intervention compliance and consequently, enhancement of general pet health.

The present study highlighted a relatively low knowledge of heartworm prevention by veterinary students despite experience in veterinary settings. To improve prevention messaging in veterinary medicine, we support interventions combining classical prevention campaigns and innovative communication tools, involving entire veterinary clinical teams and allowing the involvement of the pet owner and the use of communication tools such as social media engagement. Improving intervention messaging should start at the veterinary school and veterinary technician programmes. Attitudes in communication did not changed during the study, highlighting the importance of strengthening the education of clinical communication skills in veterinary curriculums.

## CONFLICT OF INTEREST

The authors declare no conflict of interest.

## ETHIC STATEMENT

The ethic approvals were granted by the institutional review board of RUSVM (IRB protocol #17‐10‐XP)

## Supporting information


Supporting Information 1
Standardised questionnaire used for the knowledge, attitudes and practice.

Supporting Information 2
Assessment of heartworm incidence in the USA by state in 2007 and 2016. Assessment is based on the incidence maps provided by American Heartworm Society (www.heartwormsociety.org/veterinary‐resources/incidence‐maps). A state that observed at least one area with a minimum of 26 cases by clinic was classified as a state with a high heartworm incidence (code: 1).

## Data Availability

Data available on request due to privacy/ethical restrictions.
