# Supplementary material for: Dirofilaria immitis prevention messaging: Knowledge and attitudes of students from North America attending the Ross University School of Veterinary Medicine, St Kitts
Source: Vet Rec Open. 2022 Apr 9;9(1):e32. doi: 10.1002/vro2.32 (PMC8994136; doi:10.1002/vro2.32)
Supplement: Supplementary file 1 — Supporting Information 1 Standardised questionnaire used for the knowledge, attitudes and practice. [file VRO2-9-e32-s002.pdf]

This questionnaire asks you to assess your knowledge, attitudes and practices towards an animal disease called heartworm. This questionnaire is not a test and will not be graded. You will not be judged on your answers. Please answer honestly to all questions.

This questionnaire includes 2 sections:

1) Background data and knowledge. Please complete first and turn in. The facilitator will then give you the second section to complete.

2) Attitudes and practices.

Please include the ID present on the consent form for the questions "Unique ID"

Section 1: Background Data

Unique ID:

Gender: □ Female □ Male

Age: _______________ in years

1) What is your hometown : ______________________________
   (or the town where you have spent most of your life)

   State: ________________________

   Country: ________________________

2) Town where you lived during the year before you arrived in St Kitts: _________________________

   State: ________________________

   Country: ________________________

3) Current Semester: □ Vet Prep □ 1st semester

   □ 3rd semester □ 4th semester

Unique ID:

4) Do you own an animal(s) in St Kitts? □ Yes □ No

If yes, please complete the following table:

	Owned	Number	Did the animal(s) move with you from home to St Kitts or is it from St Kitts? (if several animals, tick all relevant)
Bird	□		□ Home □ St Kitts □ Other country: Specify:_____________
Cat	□		□ Home □ St Kitts □ Other country: Specify:_____________
Cattle	□		□ Home □ St Kitts □ Other country: Specify:_____________
Dog	□		□ Home □ St Kitts □ Other country: Specify:_____________
Goat	□		□ Home □ St Kitts □ Other country: Specify:_____________
Horse	□		□ Home □ St Kitts □ Other country: Specify:_____________
Rabbit	□		□ Home □ St Kitts □ Other country: Specify:_____________
Rodent (rat/mice)	□		□ Home □ St Kitts □ Other country: Specify:_____________
Sheep	□		□ Home □ St Kitts □ Other country: Specify:_____________
Snake	□		□ Home □ St Kitts □ Other country: Specify:_____________
Tortoise	□		□ Home □ St Kitts □ Other country: Specify:_____________
Other	□		□ Home □ St Kitts □ Other country: Specify:_____________

5) Do you own an animal back home? □ Yes □ No

If yes, please complete the following table:

	Owned	Number
Bird	□	
Cat	□	
Cattle	□	
Dog	□	
Goat	□	
Horse	□	
Rabbit	□	
Rodent (rat/ice)	□	
Sheep	□	
Snake	□	
Tortoise	□	
Other	□	

Unique ID:

6) Have you ever worked in a veterinary setting before (hospital, clinic…)?

□ Yes □ No

If yes: Where was the veterinary setting located?

Town:_________________________

State: _________________________

Country: _______________________

If yes: What was your position (tick all relevant)?

□ Veterinary technician □ Veterinary assistant

□ Office assistant □ Trainee/Volunteer

□ Other (Specify: _____________________________________)

If yes: How long in total have you worked?

□ Less than one year □ 3-5 years

□ 1-3 years □ 5-10 years

□ More than 10 years

7) Where do you live in St Kitts? RUSVM Dorm □ Yes □ No

Town/Area:

Parish (please ask the facilitator or look at the map if you do not know)

□ St Mary □ St Peter □ St Paul □ Trinity □ St Anne

□ St George □ St Thomas □ St John □ Christ Church

Unique ID:

Section 1 (cont'd): Knowledge

8) Have you ever heard about heartworm disease before? □ Yes □ No

If yes: How do you know about heartworm?

□ I have/had an animal who was diagnosed with heartworm

□ I know somebody whose animal was diagnosed with heartworm

□ I have/had an animal and the veterinarian educated me about heartworm

□ I worked in a veterinarian clinic

□ Other source, describe: ____________________________________

9) Do you know the scientific name of the agent? □ Yes □ No

If yes, what is it?: _______________________________

10) Do you know which type of agent it is: □ Bacteria □ Fungi □ Parasite □ Virus

□ Don't know □ Other (Specify:__________________)

11) What is the main animal infected by this agent? ___________________

12) Does the agent infect other animals? □ Yes □ No

If yes, which ones? ____

____

13) Can the agent infect humans? □ Yes □ No

14) How is the agent transmitted?

□ Direct contact □ Indirect contact (via environment) □ By a vector □ Don't know

Unique ID:

15) Can you describe the transmission?

____

____

____

16) Can you describe the clinical signs?

____

____

____

17) Can you describe the method to prevent heartworm?

____

____

____

The first section of the questionnaire is complete. Please ask the facilitator for the second section of the questionnaire.

Unique ID:

Section 2: Attitudes

Please answer your level of agreement on the following statements:

1) Heartworm is an important disease in ***dogs*** in **USA/Canada**

□ Strongly disagree □ Moderately disagree □ Neutral □ Moderately agree □ Strongly agree

2) Heartworm is an important disease in ***cats*** in **USA/Canada**

□ Strongly disagree □ Moderately disagree □ Neutral □ Moderately agree □ Strongly agree

3) Heartworm is an important disease in ***dogs*** in **St Kitts and Nevis**

□ Strongly disagree □ Moderately disagree □ Neutral □ Moderately agree □ Strongly agree

4) Heartworm is an important disease in ***cats*** in **St Kitts and Nevis**

□ Strongly disagree □ Moderately disagree □ Neutral □ Moderately agree □ Strongly agree

5) Local dogs from **St Kitts and Nevis** are more resistant to heartworm infection compared to imported ***dogs***.

□ Strongly disagree □ Moderately disagree □ Neutral □ Moderately agree □ Strongly agree

6) There is a good and safe method to prevent heartworm in ***dogs***.

□ Strongly disagree □ Moderately disagree □ Neutral □ Moderately agree □ Strongly agree

7) I advise my neighbour/my family/my friend, who have a ***dog(s)*** in **USA/Canada** to give it preventative against heartworm

□ Never □ Sometimes □ Neutral □ Often □ Always

8) I give information on dog general health to my neighbour/my family/my friend, who have a ***dog(s)*** in **USA/Canada**

□ Never □ Sometimes □ Neutral □ Often □ Always

Unique ID:

9) I advise my neighbour/my family/my friend who have a ***dog(s)*** in **St Kitts** to give it preventative against heartworm

□ Never □ Sometimes □ Neutral □ Often □ Always

10) I give information on dog general health to my neighbour/my family/my friend, who have a ***dog(s)*** in **St Kitts**

□ Never □ Sometimes □ Neutral □ Often □ Always

11) How often should the prevention be given to ***dogs***?

□ Daily □ Weekly □ Monthly □ Every 2 months □ Every 6 months □ No opinion

12) I advise my neighbour/my family/my friend, who have a ***cat(s)*** in **USA/Canada** to give it preventative against heartworm

□ Never □ Sometimes □ Neutral □ Often □ Always

13) I give information on cat general health to my neighbour/my family/my friend, who have a ***cat(s)*** in **USA/Canada**

□ Never □ Sometimes □ Neutral □ Often □ Always

14) I advise my neighbour/my family/my friend who have ***a cat(s)*** in **St Kitts** to give it preventative against heartworm

□ Never □ Sometimes □ Neutral □ Often □ Always

15) I give information on cat general health to my neighbour/my family/my friend, who have a ***cat(s)*** in **St Kitts**

□ Never □ Sometimes □ Neutral □ Often □ Always

16) How often should the prevention be given to ***cats***?

□ Daily □ Weekly □ Monthly □ Every 2 months □ Every 6 months □ No opinion

17) I have more worries about heartworm now that I am living in St Kitts

□ Strongly disagree □ Moderately disagree □ Neutral □ Moderately agree □ Strongly agree

Unique ID:

Section 2 (cont'd): Practices (if dog owner in St Kitts only)

Please answer the following questions if you own a dog in St Kitts

1) Do you protect your dog against mosquitoes? ◻ Yes ◻ No

If yes, please describe how:____

2) Do you use any heartworm preventative medicine? ◻ Yes ◻ No

If yes, which product?____

If yes, how often? ◻ Daily ◻ Weekly ◻ Monthly

◻ Every 2 months ◻ Every 6 months

◻ When I think about it

3) Where do you keep your dog most of the time?

◻ Always inside ◻ Inside with access to the garden during the day

◻ Outside with fence ◻ Free roaming

4) Do you walk your dog outside your flat/house? ◻ Yes ◻ No

If yes, where (tick all applicable)?

◻ Around my house

◻ In the village/parish where I live

◻ Around RUSVM

◻ At the beach in frigate bay/peninsula

◻ On the golf course

◻ Other; Specify:________________________

5) Has your dog ever been diagnosed with heartworm? ◻ Yes ◻ No

If yes, was it diagnosed in St Kitts? ◻ Yes ◻ No

If yes, did it change your practices toward it? ◻ Yes ◻ No
